# Validating the Mini Sarcopenia Risk Assessment (MSRA) in Sarcopenia Case-finding for Healthy Older Adults

**DOI:** 10.1093/geroni/igaf122.2418

**Published:** 2025-12-31

**Authors:** Shiyun Chua, Wee Shiong Lim, Jia Qian Chia, Justin Chew

**Affiliations:** Tan Tock Seng Hospital, Singapore, Singapore; Tan Tock Seng Hospital, Singapore, Singapore; Tan Tock Seng Hospital, Singapore, Singapore; Tan Tock Seng Hospital, Singapore, Singapore

## Abstract

AWGS 2019 consensus emphasizes screening for early identification of people at risk for sarcopenia. The short (MSRA-5) and long (MSRA-7) questionnaires were validated in Italy, but performed poorly in diagnostic accuracy among heathy older persons in Singapore. Our study aims to examine the predictive validity of MSRA in Singapore. We performed a post-hoc secondary analysis of GeriLABS-2, a prospective cohort study of 230 community-dwelling adults ≥50 years in Singapore, at baseline and Year 3. We evaluated predictive validity of MSRA for activity participation (Frenchay Activities Index, IPAQ, Life Space Assessment), patient-reported quality of life (EQ5D), and healthcare utilization (falls, ED, hospital admissions) at Year 3 using linear and negative binomial regression. Adjusted, there was no significant association between MSRA-5 and FAI (β = -0.02 (-0.08 – 0.04)), IPAQ (β = -2.85 (-47.91 – 42.20)), LSA (β = 0.02 (-0.27 – 0.30)), or EQ5D (β = 0.08 (-0.12– 0.28)). Adjusted odds ratio for composite outcome of ≥ 1 falls, ED or hospital admission was 1.03 (0.98 – 1.09). Compared to those negative in MSRA-5, adjusted relative risk is similar for falls (0.99 (0.95-1.02)), ED (1.04 (0.99-1.11)) and hospital admission (1.06 (0.99-1.15)). Results were similar for MSRA-7, indicating poor predictive validity for activity participation, quality of life and healthcare utilization at 2 years. Overall, MSRA demonstrated poor predictive validity for a comprehensive range of clinically relevant outcomes. Taken together with the low diagnostic accuracy in an earlier study, these results highlight the limited clinical utility of MSRA in healthy Asian population.

